# Toxoplasma gondii Matrix Antigen 1 Is a Secreted Immunomodulatory Effector

**DOI:** 10.1128/mBio.00603-21

**Published:** 2021-05-18

**Authors:** Tadakimi Tomita, Debanjan Mukhopadhyay, Bing Han, Rama Yakubu, Vincent Tu, Joshua Mayoral, Tatsuki Sugi, Yanfen Ma, Jeroen P. J. Saeij, Louis M. Weiss

**Affiliations:** a Department of Pathology, Albert Einstein College of Medicine, New York, New York, USA; b Department of Pathology, Microbiology and Immunology, School of Veterinary Medicine, University of California, Davis, Davis, California, USA; c Department of Medicine, Albert Einstein College of Medicine, New York, New York, USA; Washington University School of Medicine

**Keywords:** *Toxoplasma gondii*, dense granule protein, inflammasome

## Abstract

Our studies on novel cyst wall proteins serendipitously led us to the discovery that cyst wall and vacuolar matrix protein MAG1, first identified a quarter of a century ago, functions as a secreted immunomodulatory effector. MAG1 is a dense granular protein that is found in the parasitophorous vacuolar matrix in tachyzoite vacuoles and the cyst wall and matrix in bradyzoite vacuoles. In the current study, we demonstrated that MAG1 is secreted beyond the parasitophorous vacuole into the host cytosol in both tachyzoites and bradyzoites. Secretion of MAG1 gradually decreases as the parasitophorous vacuole matures, but prominent MAG1 puncta are present inside host cells even at 4 and 6 days following infection. During acute murine infection, Δ*mag1* parasites displayed significantly reduced virulence and dissemination. In the chronic stage of infection, Δ*mag1* parasites generated almost no brain cysts. To identify the mechanism behind the attenuated pathology seen with Δ*mag1* parasites, various immune responses were screened *in vitro* using bone marrow-derived macrophages (BMDM). Infection of BMDM with Δ*mag1* parasites induced a significant increase in interleukin 1β (IL-1β) secretion, which is a hallmark of inflammasome activation. Heterologous complementation of MAG1 in BMDM cells prevented this Δ*mag1* parasite-induced IL-1β release, indicating that secreted MAG1 in host cytosol dampens inflammasome activation. Furthermore, knocking out GRA15 (an inducer of IL-1β release) in Δ*mag1* parasites completely inhibited all IL-1β release by host cells following infection. These data suggest that MAG1 has a role as an immunomodulatory molecule and that by suppressing inflammasome activation, it would favor survival of the parasite and the establishment of latent infection.

## INTRODUCTION

Toxoplasma gondii is an obligately intracellular parasite that chronically infects up to a third of the human population (1). It can cause fatal encephalitis in AIDS patients and intellectual disabilities in congenital infection. Reactivation of latent infection is an important mechanism underlying disease in both of these patient populations. Although effective commercially available drugs exist to control tachyzoites, the acute proliferative form of this parasite, there is no drug that eliminates latent tissue cysts, containing bradyzoites. Properly activated CD8^+^ cytotoxic T cells have been demonstrated to eliminate cysts in murine infections (2); however, in most infections the majority of cysts persist, and few or no inflammatory cells are seen surrounding cysts within the brain (3). Cysts are surrounded by a highly modified parasitophorous vacuolar membrane (PVM) termed the cyst wall. The cyst wall is important in the survival of cysts during transmission as well as within the central nervous system (CNS). How the cyst escapes immune surveillance is an important question that underlies the ability of T. gondii to cause chronic infections.

Recent studies have demonstrated that several dense granule proteins (GRAs) that are either present in the interface between the parasitophorous vacuole (PV) and host cytosol or secreted into the host cell modulate innate immune responses in tachyzoites. Some GRAs have immunosuppressive roles; e.g., GRA7 complexes with pseudokinases and deactivates immunity-related GTPase a6 (IRGA6), GRA18 is secreted into host cytosol and induces anti-inflammatory cytokines (CCL17/22) (4), and both TgIST and TEEGR/HCE1 translocate into the host cell nucleus, dampening the gamma interferon (IFN-γ) response (5) and NF-κB activation, respectively (6). In contrast, several other GRAs induce proinflammatory responses. For example, GRA6 activates nuclear factor of activated T cell 4 (NFAT4), a transcription factor, resulting in monocyte and neutrophil recruitment (7); GRA16 and GRA24 translocate into host nucleus and alter host gene expression, resulting in proinflammatory cytokine and chemokine production (8, 9); GRA35 activates pyroptosis in Lewis rat macrophages (10); and GRA15 from a T. gondii type II strain can stimulate inflammasome formation via NOD (nucleotide-binding oligomerization domain)-like pattern recognition receptor 3 (NLRP3) and induce interleukin 1β (IL-1β) secretion (11–13).

The inflammasome is a proinflammatory innate immune multimeric signaling complex composed of NOD-like pattern recognition receptors, an adapter protein apoptosis-associated speck-like protein containing a CARD (ASC), and caspase-1, resulting in proteolytic cleavage and secretion of proinflammatory cytokines IL-1β and IL-18 (14). The key cytokine of inflammasome activation, IL-1β, protects mice from lethal T. gondii infection (15), and mice lacking the IL-1 receptor have much higher mortality than wild-type (WT) mice when challenged with T. gondii (16). Therefore, inflammasome activation is critical for parasite control during acute infection. The diversity of the mechanisms as well as the balance of both pro- and anti-inflammatory modulation by various parasite proteins demonstrates that the T. gondii infection is the result of an intricate host-pathogen relationship. While previous studies focused on the tachyzoite stage in acute infection, we recently demonstrated that the secreted effector TgIST is exported into the host nucleus even during the cyst stage (17). However, information on the mechanism(s) of the immunomodulation of host cells by cysts during chronic infection is still lacking.

While screening a hybridoma library raised against mouse brain cysts (18), we fortuitously identified an antibody that recognized matrix antigen 1 (MAG1). Initially described a quarter of a century ago as a cyst matrix protein (19), MAG1 is a dense granular protein with no known homology to other proteins. A proteomic study of purified cyst walls demonstrated that MAG1 is an abundant component of the cyst wall (20). A proximity labeling study determined that MAG1 was part of an interactome of a group of dense granule proteins, e.g., CST1, CST2, CST9, GRA6, MCP4, MAG2, and potentially GRA7, GRA9, and GRA12 (21). This interactome analysis identified MAG1 as a hub protein that is probably critical for cyst wall composition. Other proximity labeling studies also identified additional GRA proteins (GRA1 [22], GRA13, GRA17, GRA25 [23], and GRA55 through GRA59 [24]) as interacting partners, further illustrating the role of MAG1 as a hub in cyst wall composition. A large-scale *in vivo* CRISPR knockout screen identified MAG1 as a key protein necessary for either dissemination to the brain or establishment of brain cysts (25). MAG1 is also immunologically relevant in chronic infection in both mice and humans. The level of the anti-MAG1 antibody in serum has been found to correlate with the brain cyst burden in mice (26) and, therefore, has been used in human studies as a surrogate marker for brain cyst burden (27, 28).

In this study, we demonstrate that MAG1 is secreted beyond the PV into the host cytosol in both tachyzoites and bradyzoites. In the murine infection model, Δ*mag1* parasites displayed a significantly reduced expansion and formed 98% fewer cysts in the CNS than WT parasites. In mouse bone marrow-derived macrophages (BMDM), infection with Δ*mag1* parasites induced a significant increase in IL-1β secretion, which is a hallmark of inflammasome activation. Heterologous expression of MAG1 in BMDM prevented this Δ*mag1* parasite-induced IL-1β increase, indicating that secreted MAG1 dampens the inflammasome activation in the host cytosol. These data suggest that MAG1 has a role as an immunomodulatory molecule and that by suppressing inflammasome activation, MAG1 would favor the survival of this parasite and the establishment of a latent infection.

## RESULTS

### Isolation of MAG1-specific monoclonal antibody.

**FIG 1:**
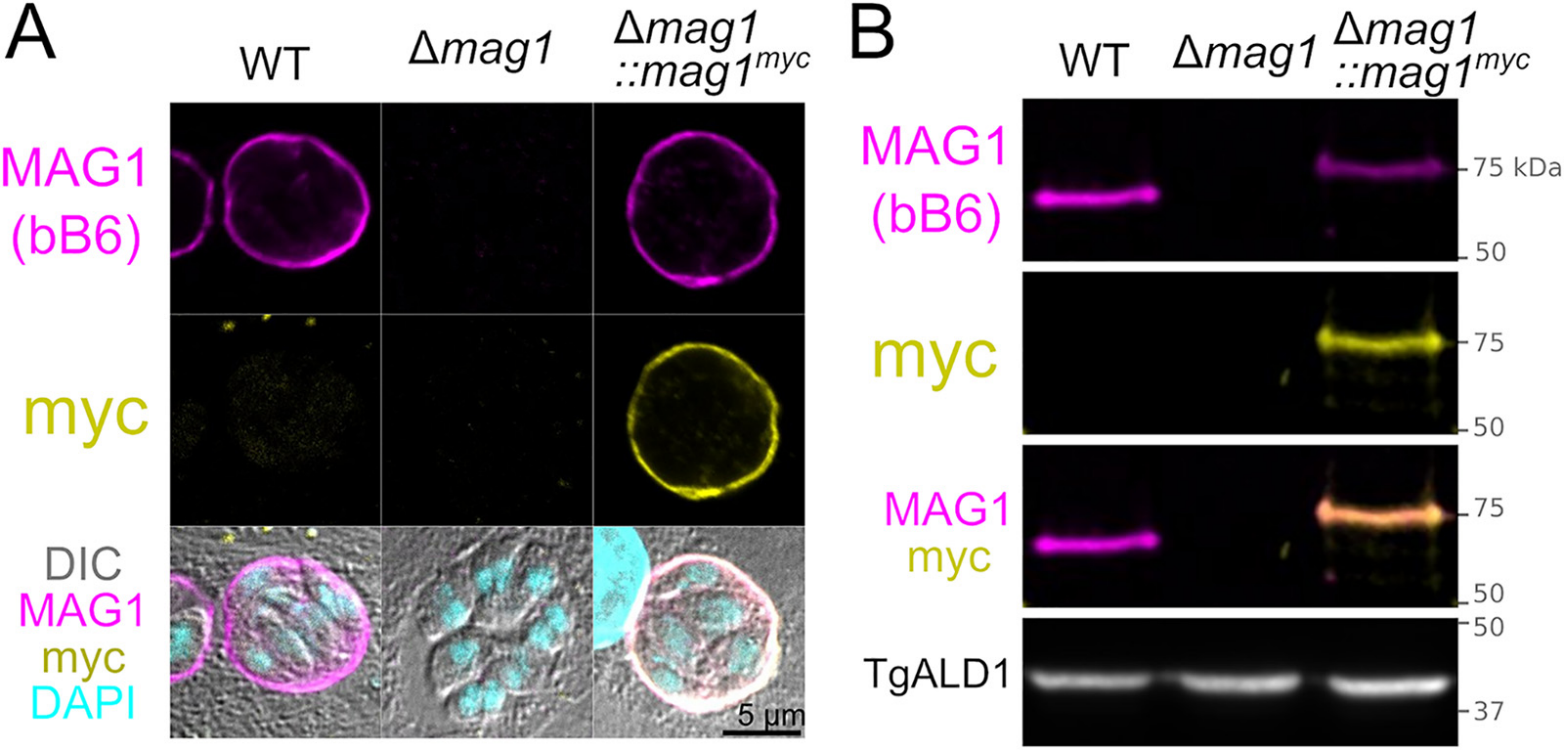
Cyst wall-specific monoclonal antibody bB6 binds to MAG1. (A) Confocal images of *in vitro* cysts at 2 dpi probed with monoclonal antibody bB6 and Myc tag antibody. Δ*mag1* parasites lose cyst wall staining, and Δ*mag1*::*mag1^myc^* complementation restores the staining. The Myc tag signal colocalizes with bB6, indicating that bB6 is binding to MAG1. (B) Immunoblot analysis of bradyzoite culture lysate probed with bB6 and Myc tag antibodies. The Δ*mag1* parasite lost the bB6-specific 65-kDa band, and the Δ*mag1*::*mag1^myc^* parasite regained the band. The Myc tag signal also binds to the restored band. T. gondii aldolase 1 (TgALD1)-specific antibody was used as a parasite-specific loading control.

To identify novel cyst wall proteins, a hybridoma library was produced by immunizing BALB/c mice with purified brain cysts from ME49 T. gondii. These antibodies were screened against *in vitro* cysts by an immunofluorescent assay (IFA) to evaluate the localization of their cognate antigens. Among the isolated monoclonal antibodies (MAbs), clone bB6 localized to the *in vitro* cyst wall of PruΔ*ku80* parasites (Fig. 1A). Immunoblot analysis of *in vitro* cyst lysate revealed that MAb bB6 reacted with a single band at 65 kDa (Fig. 1B). A cognate parasite antigen was identified by mass-spectrometric analysis of MAb bB6-immunoprecipitated lysate (see Fig. S1A in the supplemental material) as MAG1 (TGME49_270240). MAG1 localized to the cyst wall of WT parasites uniformly (Fig. 1A). To confirm that MAG1 is the target antigen of MAb bB6, the MAG1 gene (*mag1*) was deleted from the genome of T. gondii in strain PruΔ*ku80* (29) by homologous recombination, creating a Δ*mag1* T. gondii strain. A complemented strain was generated by inserting a cDNA copy of *mag1* with a C-terminal Myc tag into the UPRT locus by homologous recombination (Δ*mag1*::*mag1^myc^*). Cyst wall staining and the 65-kDa band are absent in the Δ*mag1* strain and restored in the complemented Δ*mag1*::*mag1^myc^* strain (Fig. 1A and B). Anti-Myc antibody colocalized with MAG1 signal in IFA and immunoblotting in the complemented strain. For additional confirmation, a rabbit polyclonal antibody raised against MAG1 was used to probe the lysate, which detected the same 65-kDa band (Fig. S1B).

10.1128/mBio.00603-21.1 FIG S1 Immunoblots using antibodies to MAG1. (A) Immunoblot of immunoprecipitated parasite lysate probed with bB6. (B) Immunoblot of parasite lysate probed with polyclonal anti-MAG1 antibody and bB6. Download FIG S1, TIF file, 0.5 MB. Copyright © 2021 Tomita et al. 2021 Tomita et al. https://creativecommons.org/licenses/by/4.0/ This content is distributed under the terms of the Creative Commons Attribution 4.0 International license.

### MAG1 migrates from the vacuolar matrix to the cyst wall upon bradyzoite differentiation.

**FIG 2:**
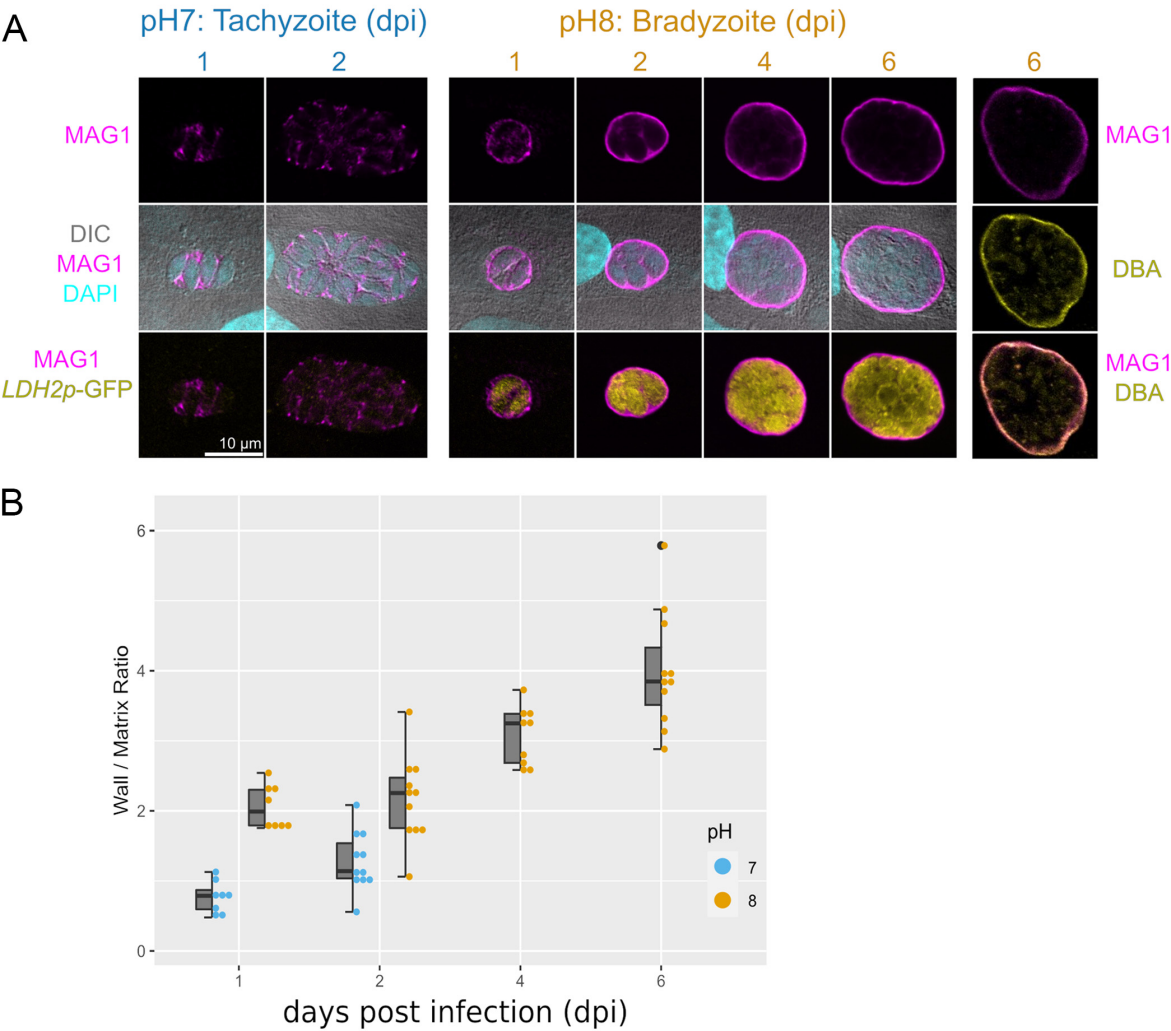
MAG1 migrates toward the cyst wall upon bradyzoite differentiation. (A) Confocal images of parasites grown in HFF were probed with MAG1- and GFP-specific antibodies. Under tachyzoite conditions (pH 7), MAG1 resides in the parasitophorous vacuolar matrix as puncta. Under bradyzoite conditions (pH 8), MAG1 migrates from the matrix to the cyst wall. GFP under the control of bradyzoite-specific marker LDH2 confirms bradyzoite differentiation. The column on the right demonstrates costaining of MAG1 and the cyst wall-specific lectin DBA, indicating that it is indeed in the cyst wall. The numbers indicate days postinfection (dpi). (B) Quantification of the wall-to-matrix ratio of mean fluorescent intensity per vacuoles.

MAG1 has been described as both a parasitophorous vacuolar matrix and a cyst wall protein (19). To determine the localization of MAG1 in the vacuolar space in the context of parasite differentiation, *in vitro* cysts were quantitatively assessed by IFA. In the tachyzoite stage, MAG1 is mostly present in the parasitophorous vacuolar matrix (Fig. 2A, left panels) with a minimal signal in the PVM at 1 and 2 days postinfection (dpi). The localization of MAG1 promptly shifts from the parasitophorous vacuolar matrix to the cyst wall upon bradyzoite induction. As differentiation progresses in pH 8 medium (indicated by GFP expression driven by the bradyzoite-specific *LDH2* promoter), the majority of MAG1 migrates to the cyst wall (located at the PVM) and the matrix staining diminishes (Fig. 2A, middle panels). Quantification of the MAG1 signal (Fig. 2B) agrees with the visual observation. To verify that the MAG1 is localized to the cyst wall, *in vitro* cysts were probed with MAb bB6 (anti-MAG1) antibody and Dolichos biflorus lectin (DBA), which binds to the *O*-GalNAc glycans on the canonical cyst wall protein CST1 (30). MAG1 and DBA colocalized in the cyst wall of *in vitro* cysts, demonstrating that MAG1 is indeed a cyst wall protein and migrates from the matrix to the cyst wall upon parasite differentiation into bradyzoites.

### MAG1 is released into the host cell.

**FIG 3:**
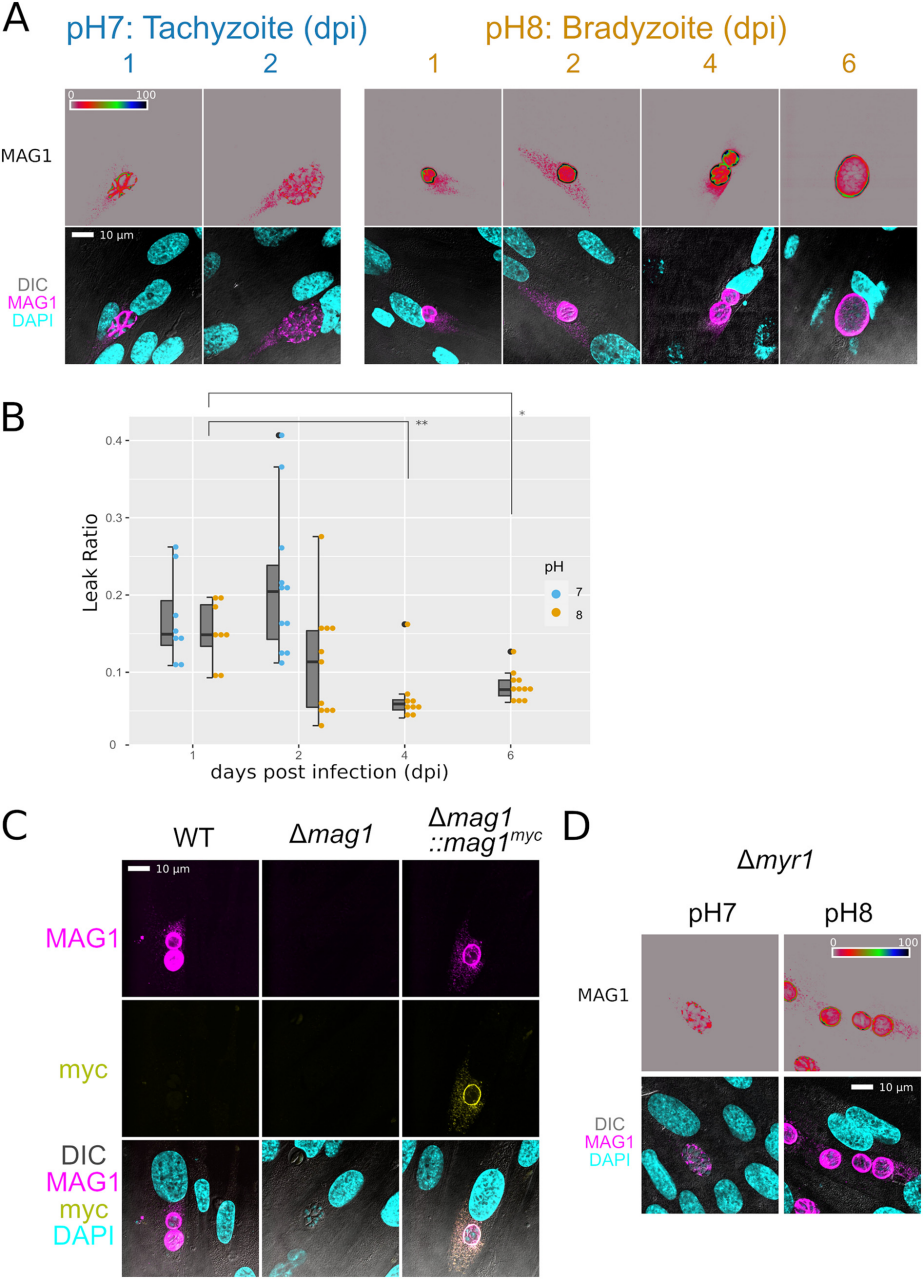
MAG1 is released into the host cell. (A) Confocal images of parasites grown in HFF that were probed with the MAG1 antibody. The intensity of MAG1 signal was displayed with high contrast in the rainbow spectrum to visualize the puncta outside the parasitophorous vacuole inside the host cytosol in both bradyzoites and tachyzoites. The puncta did not appear in uninfected neighboring host cells (lower row, with DIC and DAPI). (B) Quantification of the translocation ratio (mean fluorescence intensity of the MAG1 in host cytosol divided by total mean fluorescence). Translocation of MAG1 into the host decreases at 4 and 6 dpi (*, *P* < 0.05; **, *P* < 0.01; one-way ANOVA with Tukey’s HSD). (C) Confocal images of parasite grown in HFF probed with MAG1 and Myc tag antibodies. The translocated MAG1 is detected not only by MAG1 antibody but also by Myc tag antibody. (D) Confocal images of Δ*myr1* parasites were probed with the MAG1 antibody. The secretion of MAG1 is independent of the MYR1 translocon.

The IFA micrographic observation of MAG1 also revealed an infrequent yet conspicuous punctate signal in host cytosol in both tachyzoite and bradyzoite cell culture conditions (Fig. 3A). Fine punctate patterns inside the host cell cytosol were observed in infected host cells but not in uninfected cells, demonstrating the specificity of this MAG1 signal. Quantification of the cytosolic MAG1 demonstrated that cytoplasmic localization of MAG1 was observed starting early in infection. Although the frequency and amount of observed cytoplasmic MAG1 gradually diminished as infection progressed, there were visible punctate signals even at 4 and 6 dpi (Fig. 3A and B). To provide additional evidence for MAG1 translocation into the host cell cytoplasm, Δ*mag1*::*mag1^myc^* parasites were also probed with anti-Myc tag antibody and MAG1 antibody. C-terminally Myc-tagged MAG1 was shown to translocate into the host cell cytosol, and both the Myc and MAG1 signal colocalized (Fig. 3C). These observations suggested that MAG1 is a secreted parasite protein that might function as a secreted effector molecule.

Translocation of several GRA effectors from the parasitophorous vacuole to the host cell has been shown to be mediated by the MYR translocon. To determine if MAG1 translocation is dependent on the MYR translocon, MAG1 localization was assessed in Δ*myr1* parasites that lack the necessary component of MYR translocon. Figure 3D demonstrates that in both tachyzoite and bradyzoite conditions, MAG1 is still secreted into host cells in the absence of the MYR translocon.

### Δ*mag1* parasites have reduced virulence and fail to establish chronic infection.

**FIG 4:**
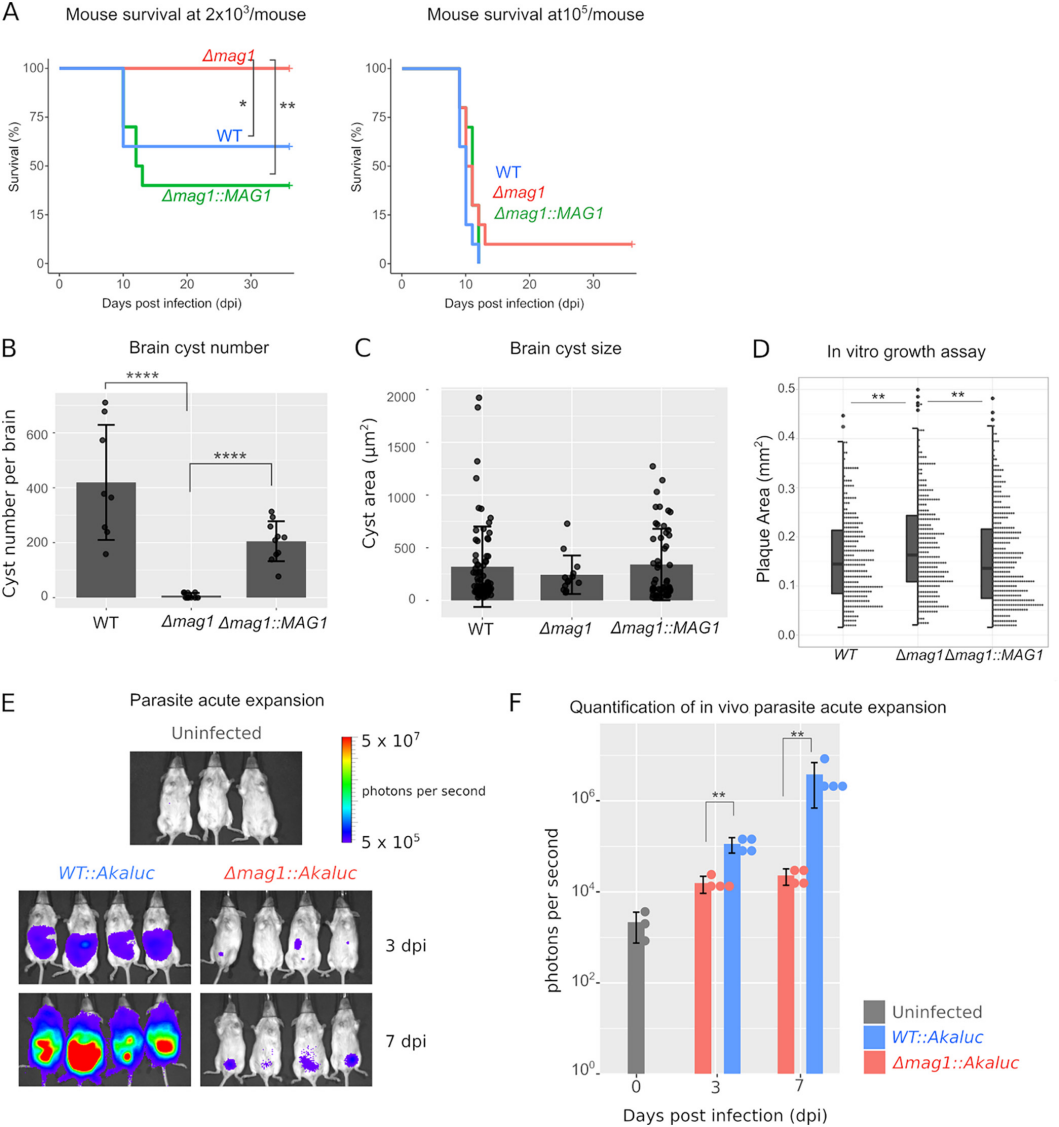
Δ*mag1* parasites have reduced virulence and fail to establish chronic infection. (A) C57BL/6 mice were infected with 2 × 10^3^ (left) or 10^5^ (right) tachyzoites i.p. (*, *P < *0.05; **, *P < *0.01; Kaplan-Meier log rank test). At a lower dosage, Δ*mag1* parasites are avirulent, but at higher dosages, they exhibit infection. (B) The brain cyst number at 35 dpi was assessed (with low inoculation dosage). Δ*mag1* parasites had much (98%) lower brain cyst numbers than the WT (****, *P < *0.0001, Kruskal-Wallis test). (C) Brain cyst size was not statistically different between WT and Δ*mag1* parasites. (D) Plaque assay was performed on parasites grown in the tachyzoite condition for 7 dpi. Δ*mag1* parasites had a small but statistically significant increase in *in vitro* growth (**, *P < *0.01; one-way ANOVA with Tukey’s HSD). (E and F) Bioluminescent imaging assay of mice infected with red-shifted luciferase-expressing parasites at 7 dpi (E) and quantification at 0, 3, and 7 dpi (F). Δ*mag1* parasites had much lower luminescence than WT parasites in mice (**, *P < *0.01; Wilcoxon rank sum test).

To investigate the function of MAG1 in the murine infection model, C57BL/6 mice (a mouse strain that is susceptible to infection) were injected with 2 × 10^3^ tachyzoites intraperitoneally (i.p.), and these infected mice were maintained until 35 dpi. While both the WT and Δ*mag1*::*mag1* parasites killed about half of the mice during this period of observation, there was no mortality in the Δ*mag1* parasite-infected mice (Fig. 4A). This suggests that the absence of MAG1 during acute infection renders the parasites avirulent at the 2 × 10^3^ inoculation dose. To determine if Δ*mag1* parasites completely lost acute virulence, mice were infected with 10^5^ tachyzoites. At this higher dosage, Δ*mag1* parasite-infected mice did succumb to infection; however, some infected mice survived infection, whereas mice infected with the WT and Δ*mag1*::*mag1* parasites did not survive beyond 10 dpi, which is consistent with a loss of virulence in the Δ*mag1* parasites.

Since MAG1 is a cyst wall protein, the effect of MAG1 deletion was assessed for brain cyst phenotypes. Surprisingly, Δ*mag1* parasites produced 98% fewer brain cysts than the WT parasites (*P < *0.0001, Kruskal-Wallis test) (Fig. 4B). While the size of the Δ*mag1* parasite brain cysts was not statistically different from sizes of the WT and Δ*mag1*::*mag1* strain cysts (Fig. 4C), large brain cysts (>1,000 μm^2^) were completely absent in Δ*mag1* strain-infected mice. Due to the paucity of Δ*mag1* brain cysts, we were not able to determine if the ultrastructure of the Δ*mag1* parasite cyst wall was altered, but cysts in tissue culture did not exhibit any obvious defects when examined under transmission electron microscopy (Fig. S2). To rule out the possibility that the absence of brain cysts was due to the impaired parasite growth, an *in vitro* plaque assay was performed. Δ*mag1* parasites had a slight, but statistically significant, increase in the plaque size (in human foreskin fibroblasts [HFF] [Fig. 4D] and mouse embryo fibroblasts [MEF] [Fig. S3A]), indicating that the brain cyst phenotype is not due to an impaired ability of the parasites to replicate.

10.1128/mBio.00603-21.2 FIG S2 Transmission electron micrograph of *in vitro* cysts. Ultrastructure of *in vitro* cysts at day 3 postinfection. B, bradyzoites; W, cyst wall. Red arrow indicates the cyst wall membrane; green arrow indicates the cyst wall granular layer. Download FIG S2, TIF file, 1.9 MB. Copyright © 2021 Tomita et al. 2021 Tomita et al. https://creativecommons.org/licenses/by/4.0/ This content is distributed under the terms of the Creative Commons Attribution 4.0 International license.

10.1128/mBio.00603-21.3 FIG S3 Immunological screening identified IL-1β secretion was elevated in Δ*mag1* parasite-infected BMDM. BMDM culture supernatant infected with parasites were assessed for (A) IL-1β, (B) TNF-α, (C) IL-12p40, (D) nitrite, (E) LDH release, (F) NF-κB activity, and (G) nuclear intensity of p65. IFN-γ susceptibility was assessed on HFF in either 10% serum (H), 10% lipid-depleted medium (I), or 1% serum (J). Δ*mag1* parasites were much more susceptible to IFN-γ only in the 1% serum medium but not in the 10% medium. Blood-circulating IFN-γ (K), IL-12 (L), and TNF-α (M) at 3 dpi were measured with parasite-infected mice. *In vivo* cytokines were not different between WT and Δ*mag1* parasites. All multiple comparisons were performed with one-way ANOVA with Tukey’s HSD test, and paired comparison was performed with a *t* test. Download FIG S3, TIF file, 0.6 MB. Copyright © 2021 Tomita et al. 2021 Tomita et al. https://creativecommons.org/licenses/by/4.0/ This content is distributed under the terms of the Creative Commons Attribution 4.0 International license.

### Acute parasite expansion is impaired in Δ*mag1* parasites.

We reasoned that the near absence of brain cysts caused by Δ*mag1* parasites could be due to a defect of cyst generation and/or failure to disseminate into the brain. To distinguish these two possibilities, parasite expansion was monitored during the acute infection in mice using a novel red-shifted bioluminescence imaging system, AkaBLI (31). WT or Δ*mag1* parasites expressing a single copy of the red-shifted luciferase Akaluc gene (32) were injected i.p. into albino C57BL6 mice (B6N-*Tyr^c-Brd^*/BrdCrCrl), and parasite expansion was monitored at day 3 and day 7 using an *in vivo* imaging system (IVIS). At both 3 and 7 dpi, infected mice had parasite luminescence in their abdomens (Fig. 4E and F). However, the intensity of the parasite signal in Δ*mag1* parasite-infected mice was reduced 86% at dpi 3 and reduced 99% at dpi 8 compared with that of the WT, indicating that MAG1 is necessary for expansion in the acute stage of mouse infection. The observations that MAG1 is secreted into the host cytosol, *in vitro* growth is not reduced in Δ*mag1* parasites, and acute expansion is significantly impaired in Δ*mag1* parasites strongly indicated that MAG1 is probably a secreted immunomodulatory factor necessary to dampen the early immune/innate response.

### MAG1 inhibits IL-1β secretion in BMDM.

**FIG 5:**
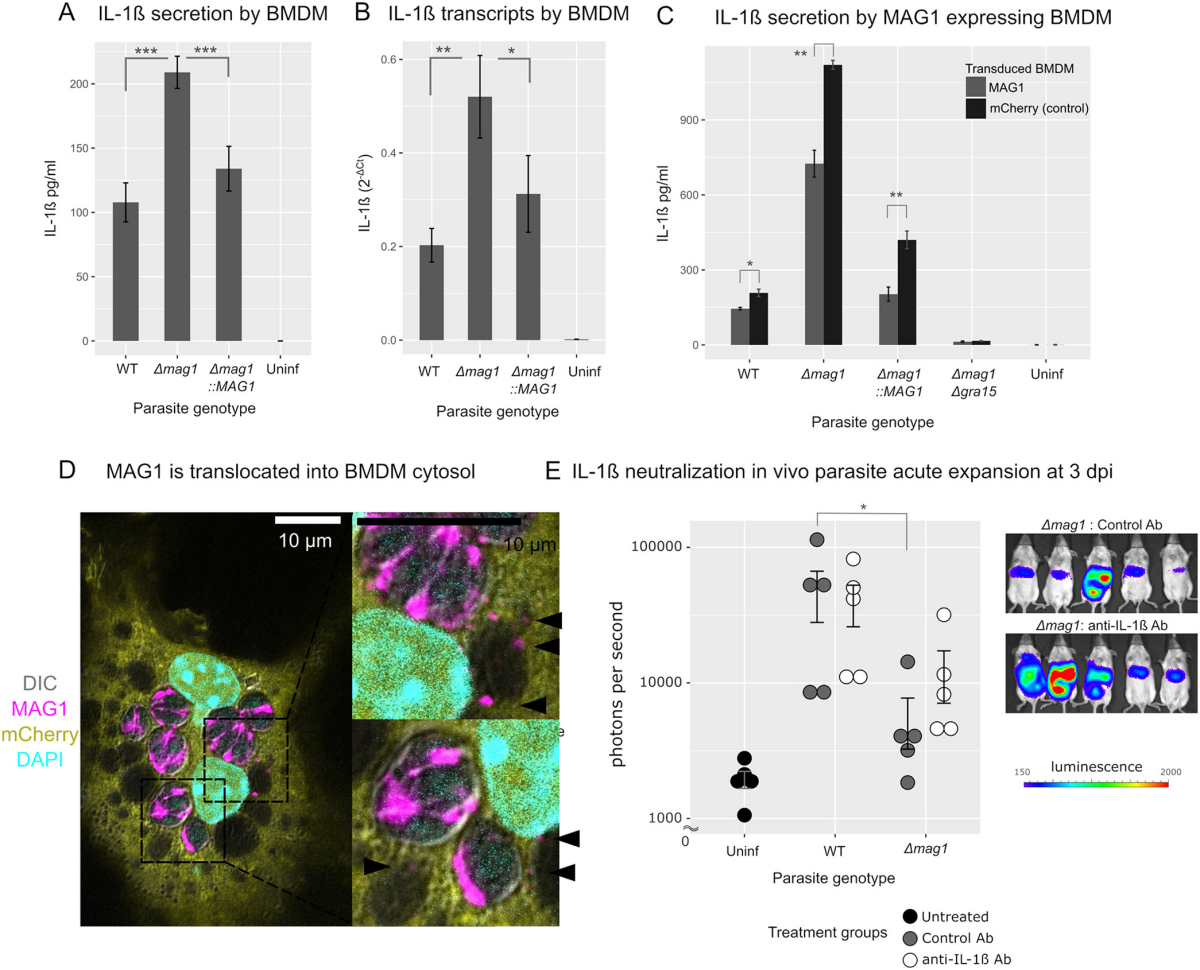
MAG1 inhibits IL-1β secretion in BMDM. (A) ELISA of IL-1β secreted by parasite infected BMDM (1 dpi, MOI = 1). Infection with Δ*mag1* parasite induced a higher release of IL-1β than that with WT and complemented strains (***, *P < *0.001; one-way ANOVA with Tukey’s HSD test). (B) The mRNA level of IL-1β was assessed in BMDM cells under the conditions used for the ELISA. Infection with Δ*mag1* parasites induced an increase in mRNA (**, *P* < 0.01; Wilcoxon rank sum test), and this was reduced in the complemented strain (*, *P* < 0.05, Wilcoxon rank sum test). (C) ELISA was performed with BMDM transduced with MAG1 or mCherry (negative control) lentivirus. Infection with Δ*mag1* parasites in MAG1-expressing BMDM is statistically lower than that in control BMDM (*, *P* < 0.05; **, *P* < 0.01; Wilcoxon rank sum test), indicating that MAG1 in the host cell is capable of dampening the IL-1β release which is induced by parasite infection. (D) IFA of BMDM transduced with mCherry lentivirus infected with WT parasite for 24 h. Black arrowheads indicate MAG1 in host cells associated with the periphery of nonparasitic vacuoles. (E) Quantification of bioluminescent imaging assay of mice infected with luciferase-expressing parasites at 3 dpi with IL-1β neutralizing antibody treatment at 1 dpi. Mice infected with Δ*mag1* parasites treated with control antibody had a significant decrease in expansion compared to mice infected with WT parasites (*, *P* < 0.05; Wilcoxon rank sum test). Mice infected with Δ*mag1* parasites treated with anti-IL-1β neutralizing antibody did not have a significant decrease in expansion compared to mice infected with WT parasites. Treatment of the Δ*mag1* strain-infected mice with anti-IL-1β neutralizing antibody increased expansion 2.2-fold compared to control antibody.

To gain mechanistic insight into the failure of the acute parasite expansion in Δ*mag1* parasites, various immune responses were screened using *in vitro* BMDM culture systems. Of the tested assays, IL-1β secretion by BMDM significantly differed from the WT (Fig. 5A and Fig. S3B), while secretion of IL-12, tumor necrosis factor alpha (TNF-α), and nitrite, NF-κB activation, IFN-γ-induced cytotoxicity, and IFN-γ susceptibility did not differ in host cells (Fig. S3C to I). Moreover, *in vivo*, circulating IFN-γ, IL-12, and TNF-α levels in blood at 3 dpi did not differ between WT and Δ*mag1* T. gondii-infected mice (Fig. S3C to M).

Although WT parasite infection induces secretion of IL-1β compared with the uninfected BMDM (Fig. 5A), infection with Δ*mag1* parasites further doubles the secretion of IL-1β compared with WT parasites (*P < *0.001; analysis of variance [ANOVA] with Tukey’s honestly significant difference [HSD] test). Complementation of the MAG1 gene reduced the level of IL-1β back to the WT level. This demonstrates that the observed IL-1β increase is dependent on MAG1. To examine if regulation was present at the transcriptomic level, the mRNA levels of IL-1β were measured (Fig. 5B). The increase in the IL-1β transcripts paralleled that of the IL-1β enzyme-linked immunosorbent assay (ELISA), suggesting that MAG1-dependent inhibition of IL-1β is also seen at the level of IL-1β mRNA.

### Heterologous expression of MAG1 in BMDM inhibits IL-1β secretion.

Since lactate dehydrogenase (LDH) release was not different between WT- and Δ*mag1* strain-infected BMDM (Fig. S3F, without IFN-γ), the pronounced IL-1β release was not due to increased cell death in Δ*mag1* strain-infected BMDM liberating cytosolic IL-1β into the medium. Rather, as MAG1 is found in both the PVM and host cytosol, its effect on IL-1β could be due to one of the following: (i) MAG1 is a major vacuolar matrix protein in the tachyzoite stage, and disruption of the vacuolar matrix affects IL-1β secretion, or (ii) MAG1 in the host cell dampens the immune response, suppressing IL-1β release. To distinguish those two possibilities, we heterologously expressed MAG1 in BMDM and assessed if heterologous complementation of MAG1 in the host cell could inhibit IL-1β secretion. This allowed us to isolate the effect of MAG1 in the host cell on IL-1β secretion. MAG1 or mCherry (negative control) genes were transduced into BMDM using lentivirus. These cells were then infected with various mutant parasites to stimulate the inflammasome (Fig. 5C). Compared with mCherry-expressing BMDM, MAG1-expressing BMDM have significantly reduced IL-1β release (*P < *0.01), indicating that MAG1 in the host cytosol is involved directly in dampening the IL-1β secretion. This reduction of the IL-1β secretion in MAG1-expressing BMDM was also observed in WT and Δ*mag1*::*mag1* parasite-infected BMDM. This suggests an additive effect of heterologously expressed MAG1 and parasite-secreted MAG1 in BMDM in suppression of IL-1β release. To confirm that MAG1 is actually secreted from the parasitophorous vacuoles into BMDM cytosol, an immunofluorescent assay was performed on the transduced BMDM (Fig. 5D). At 24 h after infection, the majority of MAG1 is present within parasitophorous vacuoles, but occasionally, MAG1 puncta were associated with host cytosol or nonparasitic vacuolar membranes (Fig. 5D, black arrowheads).

### The Δ*mag1*-induced increase in IL-1β released is dependent on GRA15.

Previous studies have demonstrated that IL-1β release is induced by a parasite-secreted effector protein, GRA15, in mouse (16) and human (12) macrophages through host inflammasome components, with a dependence on caspase 1, ASC, and NLRPs. Inflammasome activation, initiated by GRA15, is necessary for the control of acute infection in murine toxoplasmosis; thus, Δ*gra15* parasites are more virulent (13, 16, 33). In order to determine if the increase in the induction of IL-1β release in Δ*mag1* parasite infection is dependent on GRA15, we produced a T. gondii strain with a double deletion, Δ*mag1* Δ*gra15*. Our rationale was that if GRA15 is the driver of IL-1β secretion, cells infected with the Δ*mag1* Δ*gra15* strain will have no IL-1β secretion. This double-deletion mutant did not exhibit any IL-1β secretion in BMDM upon infection, demonstrating that the increase in IL-1β secretion in Δ*mag1* parasites is indeed dependent on GRA15 (Fig. 5D).

### Neutralization of IL-1β partially restores Δ*mag1* parasite virulence.

In the mouse model, the Δ*mag1* parasites have impaired acute expansion, and in the cultured macrophage model, MAG1 inhibits IL-1β secretion. We therefore examined if in murine infection with the Δ*mag1* strain, neutralization of IL-1β by antiserum during the acute phase of mouse infection could restore parasite virulence. Mice were infected with wild-type or Δ*mag1* T. gondii and then administered at 1 dpi a single i.p. injection of 10 μg of either neutralizing anti-IL-1β antibody or normal control antibody. Consistent with results in Fig. 4E, at 3 dpi following treatment with control antibody, Δ*mag1* parasites displayed a reduced parasite expansion (*P* < 0.05) (Fig. 5E) compared with WT parasites; however, there was no significant difference in parasite expansion following treatment with IL-1β antibody between WT and Δ*mag1* parasites. Treatment of Δ*mag1* strain-infected mice with just single dose of anti-IL-1β antibody increased parasite expansion 2.2-fold above that seen in the control-antibody-treated mice. This result is consistent with the hypothesis that the reduced virulence of the Δ*mag1* mutant is due to secretion of IL-1β.

## DISCUSSION

Secretion of effector GRA proteins, such as GRA16 and GRA24, into host cells is mediated by a translocon, a multimeric complex of MYR1 and MYR3 located on the PVM (34). Gradual reduction of these secreted GRAs after initial infection (17) also matches the MAG1 secretion pattern that we observed in this study (highest at 2 dpi and reduced thereafter) (Fig. 3B). However, infection of HFF with Δ*myr1* parasites still resulted in the secretion of MAG1 into the host cytosol (Fig. 3D), suggesting that MAG1 is not secreted via the MYR1 complex. Translocation of secreted GRA proteins is also dependent on proteolytic cleavage by the Golgi-residing aspartyl protease ASP5, which recognizes TEXEL motifs (35). Although MAG1 has three potential TEXEL motifs and a proteomic screening study detected cleavage ASP-5-dependent peptides (36), the immunoblot of MAG1 did not show any of the predicted ASP5-cleaved products. Taken together, these data suggest that the secretion of MAG1 is independent of both ASP5 cleavage and the MYR translocon and, thus, requires further study to uncover the yet-unknown mechanism of this effector secretion pathway.

We have demonstrated that MAG1 is released into the host cytosol by IFA. The puncta are specific to the infected host cell but are not present in uninfected host cells. An epitope-tagged version of MAG1 was also detected in the host cell using epitope-specific antibodies. This evidence strongly suggests that MAG1 secretion is not an artifact. One can still argue that MAG1 might be within parasitophorous vacuolar membrane projections or extensions (PVMP) (37, 38). However, the pattern of host cell MAG1 staining is radiating puncta around the PVM, which does not resemble the puncta on a string described for the PVMP. The punctate pattern also suggests that it might form a large complex with other host cell proteins or with other secreted parasite effectors. The molecular mechanism(s) by which MAG1 suppresses IL-1β secretion is an active area of study by our laboratory group. ELISA data demonstrated that WT parasite infection could induce some IL-1β secretion upon infection and that Δ*mag1* parasites increased the level of IL-1β compared with WT parasites. Heterologous expression of MAG1 in BMDM inhibited IL-1β release compared to infection in normal BMDM. IL-1β neutralization during acute *in vivo* infection restored expansion in the Δ*mag1* parasites but did not have an effect on parasite expansion in WT parasites. These data imply that IL-1β is involved in the reduced virulence of Δ*mag1* parasites in murine infection and that other factors may also contribute to this phenotype.

The effect of MAG1 on cyst number is striking. We reasoned that more than 98% of the reduction in the cyst burden in Δ*mag1* parasites was due to the reduction in their acute expansion based on the *in vivo* bioluminescent imaging of infected mice. We did, however, also observe a lack of large cysts in Δ*mag1* parasite-infected mice. While this was not statistically significant due to the scarcity of Δ*mag1* parasite brain cysts available for measurement, it suggests that MAG1 could also affect cyst formation. Ultrastructural studies of *in vitro*-derived Δ*mag1* parasites did not demonstrate any abnormalities in the cyst wall; unfortunately, due to the low numbers of cysts seen *in vivo*, we could not obtain any *in vivo* cysts for electron microscopy. Additional studies will be needed to discern whether MAG1 has any direct effect on cyst architecture.

**FIG 6:**
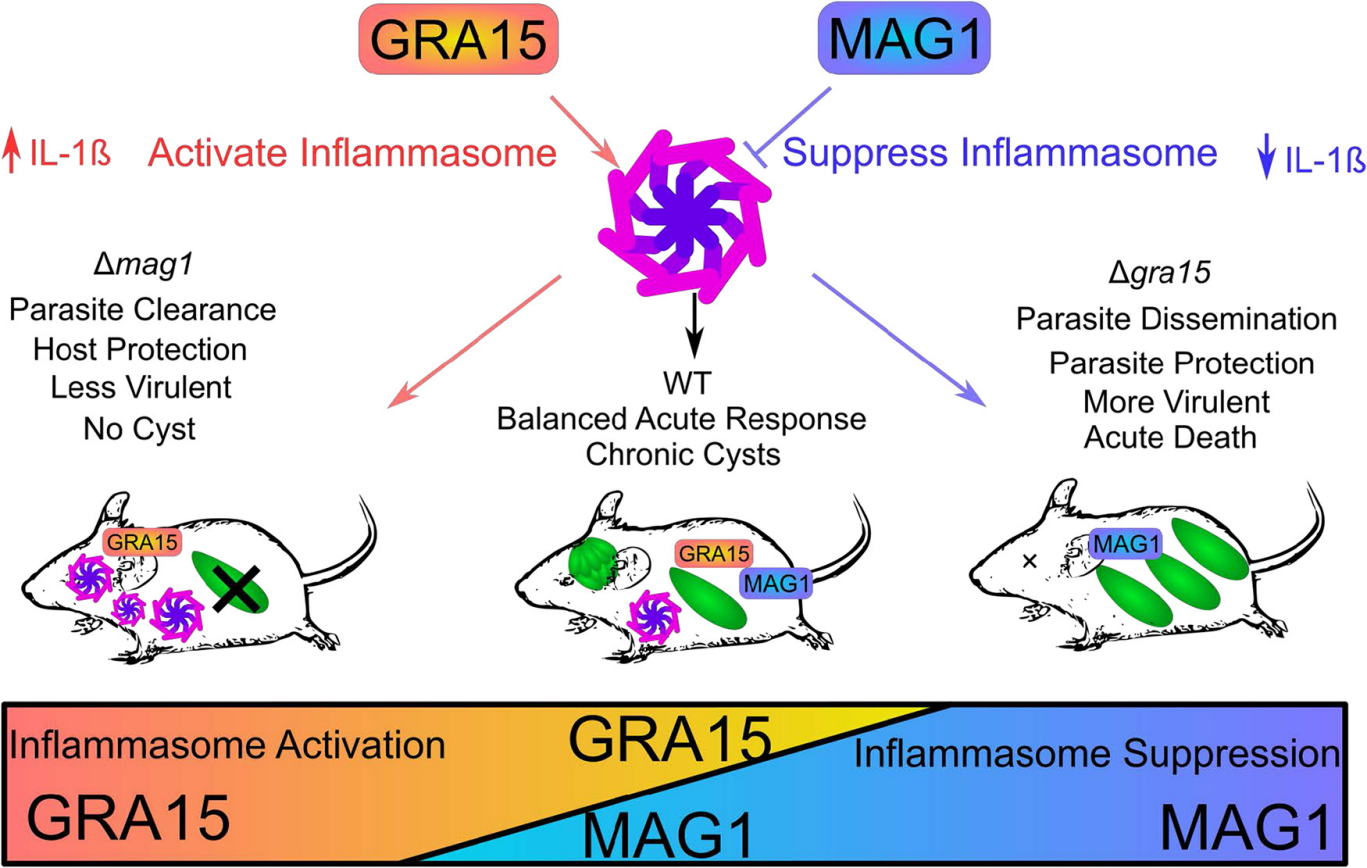
Model of MAG1 and GRA15 balancing the inflammasome response. Based on our study on MAG1 and data in the literature on GRA15, NLRPs, ASC, and caspase 1, we propose that the balance of the inflammasome response is manipulated by the antagonism of MAG1 and GRA15 during infection. GRA15 induces inflammasome activation, which protects the host during the acute stage of infection; however, MAG1 then dampens inflammasome activation, which helps the parasite disseminate and establish tissue cysts. This intricate balance of the host inflammasome response is critical for a successful chronic infection.

Previous studies of acute infection in mice with Δ*gra15* parasites demonstrated that a lack of GRA15 resulted in significantly higher acute parasite burden (11) and higher mortality (33). This suggests that activation of innate immunity by GRA15 is protective for the host animal as a means to control acute pathogenicity. In this study, we demonstrated that the Δ*mag1* parasites induced elevated IL-1β release in BMDM and exhibited a significant reduction of acute virulence and expansion. Neutralization of IL-1β partially restored this acute expansion. We propose a model of GRA15 and MAG1 activity resulting in an immunological balance for inflammasome activation in acute infection (Fig. 6). In this model, GRA15 activates the inflammasome, resulting in host protection, and MAG1 suppresses the inflammasome, resulting in parasite proliferation. In the presence of both effectors, the balance of activation/suppression enables parasites to acutely disseminate without killing the host animal (GRA15) and to subsequently establish chronic cysts (MAG1).

GRA15 activates the NF-κB pathway, which results in various types of immune activation, including activation of the inflammasome and subsequent IL-1β release. In contrast, a recently discovered dense granular protein, TEEGR (6) (also called HCE1 [39]), which translocates into the host nucleus using the MYR translocon, counteracts NF-κB activation by activating a polycomb repressive complex and subsequently leads to suppression of IL-1β release. The mechanistic difference between MAG1 and TEEGR/HCE1 is that MAG1 does not interfere with the NF-κB pathway (Fig. S3G and H) or other proinflammatory cytokines that are downstream of NF-κB (TNF-α and IL-12) (Fig. S3C and D). However, the mRNA level of IL-1β was upregulated in the *Δmag1* parasite, suggesting that the effect of MAG1 is complex. A potential point of intervention by MAG1 could be at the transcript stability stage, where MAG1 may increase the degradation of IL-1β mRNA. Alternatively, MAG1 might also interfere with the signal II pathway, which catalyzes the cleavage of pro-IL-1β for the release. Various pathogens are known to interfere with inflammasome assembly and activation, for example, by sequestering ASC or NLRPs, and to enzymatically prevent caspase-1 activity (40). If MAG1 inhibits the formation of inflammasome or processing of pro-IL-1β, deleting MAG1 could increase IL-1β secretion and released IL-1β, in turn, would bind to IL-1R and stimulate canonical NF-κB, resulting in the increase in IL-1β mRNA, which is also consistent with our observation. Further investigations seeking to identify a host factor that is targeted by MAG1 are needed to elucidate the mechanism(s) of action of MAG1 in IL-1β secretion.

In addition to GRA15 inducing NF-κB, the parasite can activate innate immune responses via GRA24, another dense granule protein, which activates the p38α mitogen-activated kinase (MAPK) pathway (8). In macrophages, both GRA15 and GRA24 synergistically activate host cells and result in proinflammatory cytokine production (IL-1β, IL-12, TNF-α, and IFN-γ) (13). We have demonstrated that the effect of MAG1 is dependent on GRA15, but whether GRA24 is involved in this process is currently unknown.

In summary, we have demonstrated that MAG1 functions as a secreted effector that modulates inflammasome activity and acts as a counterbalance to GRA15-induced inflammasome activity. The effect of MAG1 on the inflammatory response supports the development of latency. The mechanism by which MAG1 is released from the cyst into its host cell is currently unknown. MAG1 may also have direct effects on cyst development *in vivo*, but more study is needed to define its role in cyst biology.

## MATERIALS AND METHODS

### T. gondii cell culture and strains.

The type II strain Prugniaud with a deletion in the KU80 gene (PruΔ*ku80*) (29) was passaged in human foreskin fibroblasts (HFF) cultured in either 10% fetal bovine serum (Gibco) or EquaFetal (Atlasbio) in Dulbecco’s modified Eagle medium (DMEM) with penicillin-streptomycin at 5% CO_2_. For *in vitro* bradyzoite differentiation, T. gondii strains were grown in differentiation medium (DMEM medium adjusted to pH 8.1 with 25 mM HEPES and 1% fetal bovine serum with penicillin-streptomycin) at 0.5% CO_2_.

### Murine model of toxoplasmosis.

Parasites were lysed out of HFF by passage through a 27-gauge needle and filtered with a 5-μm filter. C57BL/6 mice were intraperitoneally (i.p.) injected with tachyzoites at 2 × 10^3^ to 1 × 10^5^ parasites per mouse. Viability of the parasite inoculant was verified by plaque assay. Mice were monitored daily and maintained until 35 dpi, at which point any surviving mice were euthanized. All procedures were done under an animal use protocol approved by the Albert Einstein College of Medicine IACUC.

### Monoclonal antibody production.

BALB/c^dm1^ mice (41) were infected with T. gondii strains and treated with sulfamerazine at 30 mg/liter in drinking water to minimize death from the acute infection. Four weeks after the infection, brain cysts were isolated using a previously described isopycnic centrifugation technique (42). The emulsified cyst preparation with Freund’s complete adjuvant was injected into BALB/c mice subcutaneously. Two months later, spleens were isolated from the immunized mice and fused with a myeloma cell line to create hybridoma libraries. Promising antibody candidates were screened using IFA, ELISA, and immunoblotting using WT T. gondii organisms that were cultured in pH 8 medium. For WT parasites, we found that IFA results were identical for ME49 and PruΔ*ku80* T. gondii strains. Selected monoclonal lines were then subcloned by limiting dilution to obtain clonal lines. For large-scale production of cell culture-derived monoclonal antibody, subcloned MAbs were cultured in a CELLine bioreactor (Integra) using standard techniques.

### Immunoprecipitation of the bB6 MAb antigen.

HFF were infected with PruΔ*ku80* parasites at a multiplicity of infection (MOI) of 1. Parasites were differentiated to bradyzoites and lysed in radioimmunoprecipitation assay (RIPA) buffer (50 mM Tris [pH 7.5], 150 mM NaCl, 0.1% SDS, 0.5% sodium deoxycholate, 1% NP-40). Bradyzoite lysates were precleared by incubation with protein G agarose beads for 30 min at 4°C to remove nonspecifically bound proteins. Then, precleared samples were incubated with protein G agarose beads covalently cross-linked with MAb bB6 using bis(sulfosuccinimidyl)suberate (Thermo Scientific) according to the manufacturer’s instructions. Precleared samples were incubated with MAb bB6 beads overnight at 4°C while rotating. Beads were washed twice with RIPA buffer before elution in Laemmli sample buffer. MAb bB6, lysate input, bead flowthrough, RIPA washes, and bead eluates were resolved on a 4-to-15% polyacrylamide gel (Bio-Rad) and stained with Coomassie blue. Bands in the eluate lanes were excised and analyzed by mass spectrometry.

### Mass spectrometry.

Excised gel bands were reduced, alkylated, and digested with trypsin. LC-ESI-MS/MS (liquid chromatography-electrospray ionization-tandem mass spectrometry) analysis of the peptide digests was done by C_18_ reversed-phase (RP) chromatography using an Ultimate 3000 RSLCnano system (Thermo Scientific, USA) equipped with an Acclaim PepMap RSLC C_18_ column (2 μm, 100 Å, 75 μm by 15 cm; Thermo Scientific, USA). The ultra-high-performance liquid chromatograph (UHPLC) was connected to a TriVersa NanoMate nano-electrospray source (Advion, USA) and an LTQ-XL linear ion trap (Thermo Scientific, USA) mass spectrometer with the ESI source operated in the positive ionization mode. Automated protein identification was performed by Mascot search engine v. 2.5.1 (Matrix Science) against the ToxoV12_uniprot_20150225 database (27,608 entries) with the following search parameters: trypsin, three missed cleavages, peptide charges of +2 and +3, peptide tolerance of 2.0 Da, MS/MS tolerance of 0.8 Da, carbamidomethylation (Cys) for fixed modification, and deamidation (Asn and Gln) and oxidation (Met) for variable modifications. A decoy database search was also performed to measure the false discovery rate. Mascot protein identification results were further analyzed by Scaffold software v. 4.4.5 based on 99% protein and 95% peptide probabilities.

### Production of mutant strains of T. gondii.

All MAG1 mutants except the Δ*mag1* line used in the immunological response screening were generated in the T. gondii strain Prugniaud (type II), which has a deletion of the *ku80* gene (PruΔ*ku80*) for increased homologous recombination (see Text S1). All plasmid sequences used for genetic manipulation are available in GenBank format with primers as annotations (see Data Set S1).

10.1128/mBio.00603-21.4 DATA SET S1 Plasmids and genomic sequences in GenBank format. The plasmids and genomic sequences are described using standard GenBank format with primer annotations. pMAG1KO-DHFR for Δ*mag1* generation, pMAG1-3MYC-HXGPRT for MAG1 complementation, plasmid pCas9GFP-HXGPRT-GRA15-1, the genomic locus GRA15_locus_KO1 for Δ*gra15* generation, pUPRT-GRA1-Akaluc for red-shifted luciferase-expressing parasite generation, and pHAGE-CMV-MAG1-IRES-GFP for MAG1 lentivirus production. Download Data Set S1, TXT file, 0.1 MB. Copyright © 2021 Tomita et al. 2021 Tomita et al. https://creativecommons.org/licenses/by/4.0/ This content is distributed under the terms of the Creative Commons Attribution 4.0 International license.

10.1128/mBio.00603-21.6 TEXT S1 Detailed descriptions of (A) production of mutant strains of T. gondii, (B) immune response screening, and (C) transmission electron microscopy. Download Text S1, DOCX file, 0.01 MB. Copyright © 2021 Tomita et al. 2021 Tomita et al. https://creativecommons.org/licenses/by/4.0/ This content is distributed under the terms of the Creative Commons Attribution 4.0 International license.

### Immunofluorescence assay and quantification of MAG1 cyst wall migration and host cytosol translocation.

Coverslips containing T. gondii-infected HFF were fixed with 4% paraformaldehyde in phosphate-buffered saline (PBS) for 30 min and then permeabilized with PBS containing 0.2% Triton X-100 for 20 min. The coverslips were then washed twice with 0.2% bovine serum albumin (BSA) in PBS and incubated with 1% BSA in PBS as a blocking solution at room temperature for at least 1 h. Primary antibodies were diluted in 1% BSA in PBS, and then slides were incubated with the antibody at 37°C for 90 min. bB6 antibody was used at 1:500, and mouse anti-Myc tag (Cell Signaling; 71D10) was used at 1:200. The coverslips were then washed twice with 0.2% BSA in PBS and incubated in a secondary antibody solution in 1% BSA in PBS for 37°C for 90 min, after which the coverslips were washed twice with 0.2% BSA in PBS. Coverslips were then mounted onto slides using ProLong Gold with DAPI (4′,6-diamidino-2-phenylindole; Molecular Probes) and left to dry in the dark.

For the quantification of MAG1 signal, images were taken with a Leica SP5 confocal microscope. Using ImageJ custom macros (Data Set S2), the PVM was recognized by Canny Edge Detector or, if the detection failed, annotated manually. From the PVM, a 1-μm width inward was segmented as cyst wall, and matrix thereafter. From the PVM, a 5-μm width outward was segmented as host cell. An example of a segmented image is provided in Data Set S2. MAG1 cyst wall migration was determined by the calculation of mean signal intensity in the cyst wall segment divided by the mean signal intensity in the matrix. Translocation of MAG1 was quantified by the signal in the host cell divided by the host cell, cyst wall, and matrix. One-way ANOVA with *post hoc* Tukey HSD test was used to determine the difference in the translocation of MAG1.

10.1128/mBio.00603-21.5 DATA SET S2 ImageJ macro and an example of image segmentation for MAG1 IFA. The ImageJ script was used to analyze the MAG1 intensity in the IFA images. The actual example of segmented *in vitro* cysts MAG1 images with annotated segments. Download Data Set S2, DOCX file, 0.5 MB. Copyright © 2021 Tomita et al. 2021 Tomita et al. https://creativecommons.org/licenses/by/4.0/ This content is distributed under the terms of the Creative Commons Attribution 4.0 International license.

### Plaque assay.

HFF monolayers were grown to confluence on 6-well plates and infected with 5-μm-filter-purified parasites. Parasites were allowed to grow without disturbance for 7 days. After incubation, cells were fixed, stained with a staining solution (20% methanol, 1% crystal violet in distilled water) for 30 min, and washed with water 10 times. Plaques were imaged and size was measured with ImageJ with the Trainable Weka Segmentation plugin (43). Statistical analysis was performed by one-way ANOVA with a *post hoc* test of Tukey's HSD test.

### Immunoblot analysis.

HFF infected with T. gondii were grown in DMEM medium (10% fetal bovine serum, with 1% penicillin-streptomycin) at 5% CO_2_ for tachyzoites or in differentiation medium (DMEM adjusted to pH 8.2 with 25 mM HEPES and 1% fetal bovine serum with 1% penicillin-streptomycin) at 0% CO_2_ for bradyzoites. Cells were harvested at 3 dpi lysed with RIPA buffer containing protease inhibitor cocktail. The samples were resolved by 10% SDS-PAGE and transferred using a semidry transfer system (Bio-Rad) onto Immobilon-FL (Millipore) polyvinylidene difluoride (PVDF) membranes. The membrane was blocked overnight with 5% nonfat dry milk (NFDM) in PBST and then probed with bB6 MAb at 1:500 in 1% BSA–PBS, polyclonal rabbit anti-MAG1 (gift from S. Parmley [19]) at 1: 500, or rabbit anti-Myc tag antibody. Following antibody incubation, the membrane was washed three times with PBS containing Tween (PBST) and incubated in anti-mouse IgG IRDye800 and anti-rabbit IRDye 680 at 1:40,000 in NFDM-PBST. The membranes were scanned using an Odyssey (LiCor) imaging system.

### Transmission electron microscopy.

The method used for the ultrastructural assay of *in vitro* cysts is described in Text S1.

### Immune response screening.

Immune response screening methods, including *in vitro* IFN-γ susceptibility measurement by plaque assay, measurement of NF-κB activation, LDH release assay, and *in vivo* infection for cytokine measurement, *in vitro* cytokine ELISA, and determination of nitrite, are described in Text S1.

### Preparation of BMDM and transduction with MAG1 lentivirus.

Culturing of BMDM was performed according to a previous publication (44) with modifications. Briefly, cells were isolated from bone marrows of C57BL/6 mice (day 0), RBC cells were lysed with 2-min incubation in ACK buffer, and cells were cultured in αMEM with 15% fetal bovine serum (FBS), 1 nM recombinant mouse IL-3, and 0.6 ng/ml of macrophage colony-stimulating factor (M-CSF) (M1 medium) for 3 days. At day 1, mature macrophages attached to tissue culture plates were discarded, and clusters of nonadherent precursors were dispersed with Pronase treatment and replated in M1 medium. At day 3, cells were dispersed with Pronase treatment again and replated in αMEM with 15% FBS and 120 ng/ml of M-CSF (M3 medium). MAG1 or mCherry expression lentiviruses were generated by transfection of HEK293T cells with pCMV-dR8.2, pCMV-VSV-G, and either pHAGE-CMV-MAG1-IRES-ZsGreen or pHAGE-CMV-mCherry-IRES-ZsGreen using the calcium chloride method. BMDM precursors were transduced with the lentivirus with 8 μg/ml Polybrene and incubated for 24 h. At day 4, the medium was removed and another lentivirus containing medium was added and incubated for 24 h.

### *In vivo* imaging for T. gondii dissemination in mice.

Six- to eight-week-old female albino C57BL/6 (B6N-Tyrc-Brd/BrdCrCrl; strain code 493; Charles River) mice were injected i.p. with 10^3^ freshly liberated Akaluc-expressing parasites from HFF and filtered with 5-μm filters. At 3 and 7 days after infection, mice were injected with 1.5 μmol of Tokeoni/Akalumin-HCl (Sigma-Aldrich) in 100 μl PBS i.p. and imaged at 20 min after injection using IVIS Spectrum (PerkinElmer) under anesthesia.

### IL-1β neutralization in murine T. gondii infection.

Albino C57BL/6 mice were injected i.p. with 10^3^ freshly liberated Akaluc-expressing WT and Δ*mag1* parasites as described above. At 1 dpi, mice were injected i.p. with 10 μg of either neutralizing antibodies against IL-1β or normal goat IgG (AF-401-NA and AB-108-C, respectively; R&D Systems) as previously described (45).

### ELISA and quantitative PCR in BMDM.

At 12 days postharvest, fully differentiated BMDM were seeded at 10^5^/well in 96-well tissue culture plates overnight. At day 13 (10 days after differentiation), BMDM were infected with freshly lysed parasites filtered with 5-μm membrane at an MOI of 1 to 2 (initially infected at an MOI of 1, 3, or 5 and then adjusted after plaque viability was checked). After 24 h of infection, supernatants were removed and assayed for IL-1β secretion with a mouse IL-1β/IL-1F2 DuoSet ELISA (R&D Systems; DY401) following the manufacturer's protocol.

Quantitative PCR was performed as described previously (46). Briefly, mRNA was isolated from BMDM in 96-well with a NucleoSpin RNA Mini and reverse transcribed with PrimeScript RT, and qPCR was performed with TB Green Premix *Ex Taq* II (TaKaRa) with iQ5 (Bio-Rad), following the manufacturer's protocols. Gene expression of IL-1 β was normalized against a housekeeping gene RPL27 (PrimerBank [47] ID numbers 6680415a1 and 8567400a1, respectively; sequences are in Text S1).

### Ethics statement.

All mouse experiments were conducted according to guidelines from the U.S. Public Health Service Policy on Humane Care and Use of Laboratory Animals. Animals were maintained in an AAALAC-approved facility, and all protocols were approved by the Institutional Care Committee of the Albert Einstein College of Medicine, Bronx, NY (Animal Protocol 20180602; Animal Welfare Assurance no. D16-00200).
