# Supplementary figures and images for: Toxoplasma gondii Matrix Antigen 1 Is a Secreted Immunomodulatory Effector
Source: mBio. 2021 May 18;12(3):e00603-21. doi: 10.1128/mBio.00603-21 (PMC8262993; doi:10.1128/mBio.00603-21)

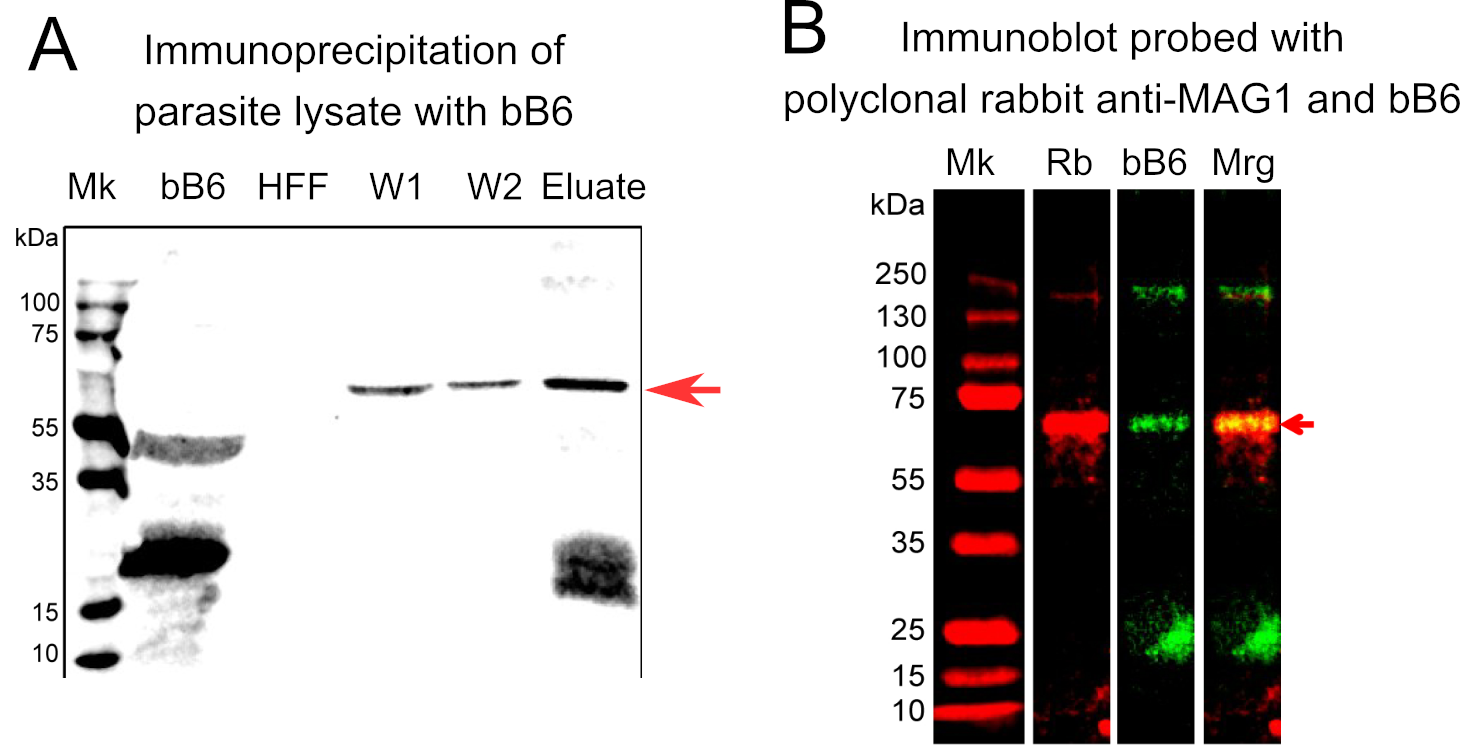

Supplement: FIG S1 [file mbio.00603-21-sf001.tif]

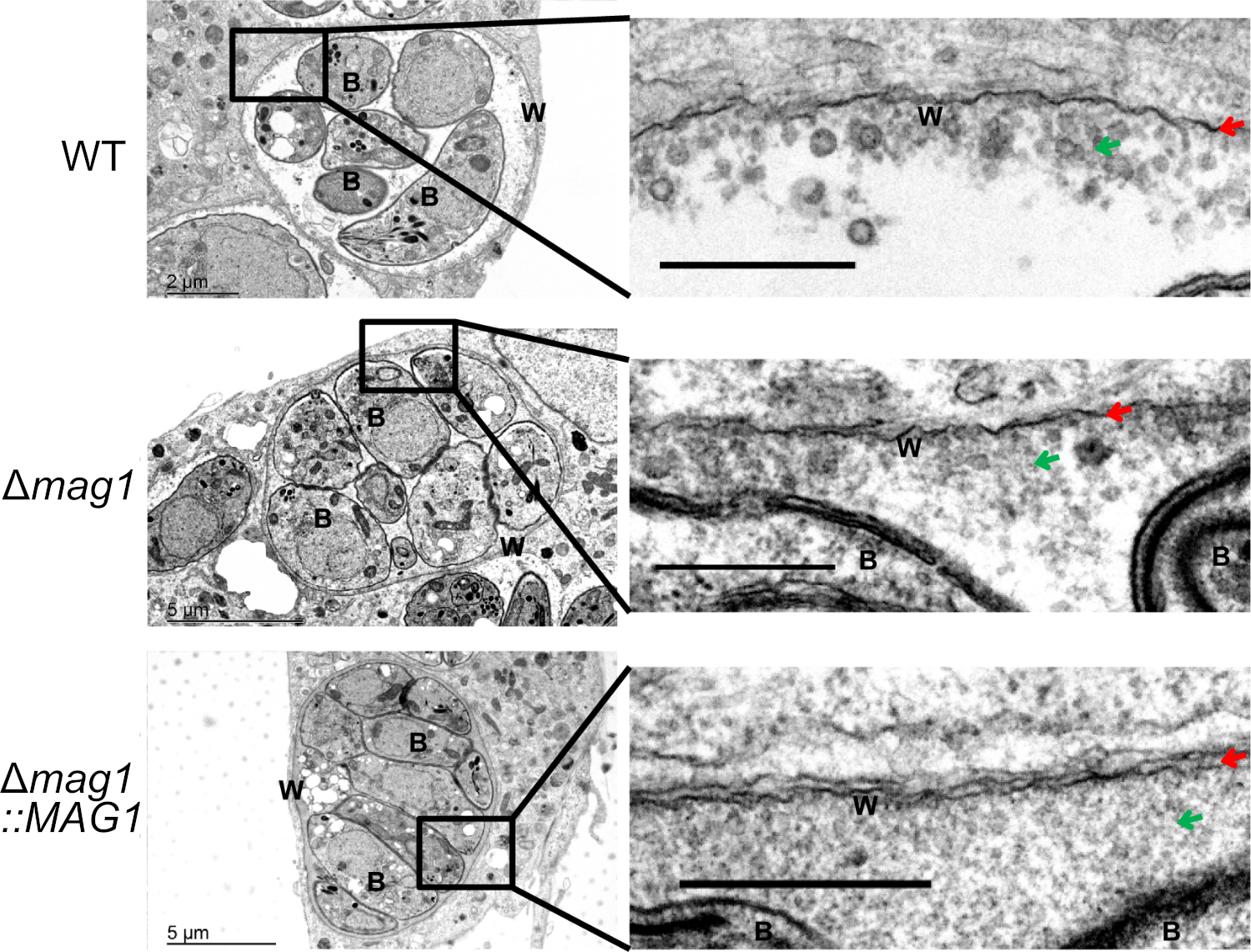

Supplement: FIG S2 [file mbio.00603-21-sf002.tif]

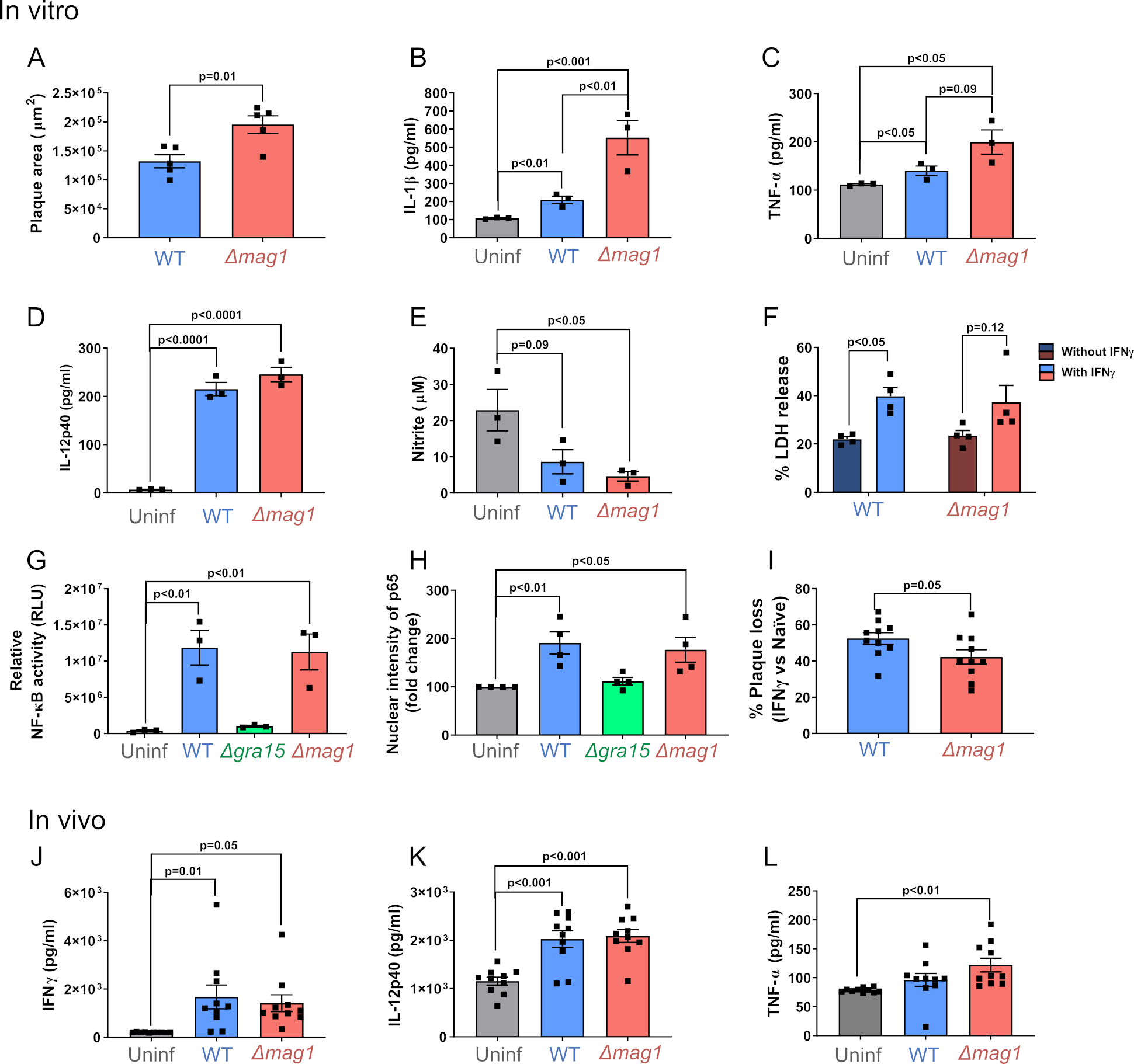

Supplement: FIG S3 [file mbio.00603-21-sf003.tif]
