# Supplementary material for: Toxoplasma gondii Matrix Antigen 1 Is a Secreted Immunomodulatory Effector
Source: mBio. 2021 May 18;12(3):e00603-21. doi: 10.1128/mBio.00603-21 (PMC8262993; doi:10.1128/mBio.00603-21)
Supplement: TEXT S1 [file mbio.00603-21-s0001.docx]

Supplementary Methods 1

## Production of mutant strains of *T. gondii*

All MAG1 mutants, excepting the *Δmag1* line used in the immunological response screening, were generated in the *T. gondii* strain Prugniaud (Type II) that has a deletion of the *Ku80* gene (Pru Δ*ku80*) for increased homologous recombination. All plasmid sequences used for genetic manipulation are available in Genbank format with primers as annotations (Supplemental file 1). All PCR amplifications were performed using Q5 polymerase, plasmid constructions were performed with the NEBuilder HiFi DNA Assembly kit (New England Biolab). All transfections of parasite were performed using 10 µg of linearized plasmid in cytomix (120 mM KCl; 0.15 mM CaCl2; 10 mM K2HPO4/KH2PO4, pH 7.6; 25 mM Hepes, pH 7.6; 2 Mm EGTA, pH 7.6; 5mM MgCl2; pH adjusted with KOH) with 1500 V 25 µF 25 Ω single exponential pulse.

Briefly, the *MAG1* locus was replaced with a DHFR selectable cassette flanked by 1 kb genomic sequence of MAG1 (TGME49_270240) homologous arms using a linearized pMAG1KO-DHFR. Transfected parasites were placed under 1 µM pyrimethamine drug selection and subcloned by limiting dilution. Deletion was confirmed by the Sanger sequence and the absence of bB6 antibody staining in cysts. Complementation of MAG1 was performed with pMAG1-3MYC-HXGPRT that contains cDNA copy of MAG1 with C-terminal 3myc tag with HXGPRT selectable marker. Transfection was performed with NotI linearized vector to Δ*mag1* strain and selected with mycophenolic acid /xanthine (25 and 50 µg/ml) for three weeks. After subcloning by limiting dilution, complementation was verified by IFA with bB6 and myc-tag specific antibody. Resultant complemented strain was designated as PruΔ*mag1::MAG1^myc^*. To generate MAG1/GRA15 double deletion mutant, pCas9GFP-HXGPRT-sgGRA15-1 vector targeting the start codon was generated with the primers specified in the Genbank file. Δ*mag1* parasites were electroporated with 10 µg of circular Cas9 plasmid and 10x molar ratio of donor oligos that contains 3x stop codons (annotated in GRA15KO_locus_KO1). Transfected parasites were selected in mycophenolic acid / xanthine for five days and subcloned by limiting dilution. Deletion of GRA15 was confirmed by PCR and Sanger sequencing. The Δ*mag1* line used for the immunological response screening was generated by the use of a clustered regularly interspaced short palindromic repeat (CRISPR)-Cas9 based system. To generate the knockout, an sgRNA sequence (the table below) against MAG1 was cloned into the pSS013-Cas9 vector, which was transfected along with NotI (New England Biolabs)-linearized pLoxp-DHFR-mCherry, which contains a pyrimethamine resistance cassette, at a ratio of 5:1 as described previously [(Niedelman et al. 2012)](https://paperpile.com/c/kMWCwc/loteh). After three rounds of pyrimethamine selection (1 µM) and limiting dilution cloning, the MAG1 knockout parasites were assessed by PCR and confirmed by sequencing.

WT and *Δmag1* parasites were transfected with a linearlized plasmid pUPRT-GRA1pAkaLuc with red-shifted luciferase Akaluc (generous gift from Atsushi Miyawaki, RDB15781 RIKEN) [(Iwano et al. 2018)](https://paperpile.com/c/kMWCwc/IMM7u) under the control of GRA1 promoter for constitutive expression. The use of this vector allows the single copy integration of Akaluc gene into UPRT locus, thus bioluminescence of various strains can be quantitatively measured and compared. Transfected parasites were selected with 5 µM of 5’fluorodeoxyuridine (FUDR) for a week and subcloned by limiting dilution. Expression of Akaluc was determined by measurement of luminescence with treatment of extracellular parasites with 500 µM of Akalumine-HCl for 20 minutes in 96-well.

**Primers used for the generation of MAG1 deletion mutant used in the immunological response screening.** Nucleotides highlighted in grey are part of the BsaI cloning site of pSS013 and nucleotides in bold are actual sgDNA sequences.

| TGME49_270240_gRNA1_Fwd | 5’ AAGTT**GGCACGGGAGCTCGACGGTC**G 3’ |
| --- | --- |
| TGME49_270240_gRNA1_Rev | 5’ AAAAC**GACCGTCGAGCTCCCGTGCC**A 3’ |
| TGME49_270240_gRNA2_Fwd | 5’ AAGTT**GTCCTTTGATGAAGTAGGCA**G 3’ |
| TGME49_270240_gRNA2_Rev | 5’ AAAAC**TGCCTACTTCATCAAAGGAC**A 3 |
| MAG1_Fwd | 5**’** GAAGTTGCAGTCACCCTTCTTGTTG 3’ |
| MAG1_Rev | 5’ GCAATAAAACGCGGATGGACTAC 3’ |
| DHFR_Rev | 5’ ATAGTCCTGTCGGGTTTCGCCAC 3’ |

## Immune Response Screening

**In vitro IFNγ-susceptibility measurement by plaque assay.** To determine the susceptibility of the MAG1 knockout parasites to IFNγ, freshly confluent 24 well plates of HFFs or MEFs were used. On the day of infection, fresh media was added replacing the old media and 250 freshly harvested parasites were added to the cells in the wells. An additional set of wells that were pre-stimulated with IFNγ (10 U/mL human IFNγ or 100 U/mL mouse IFNγ) for 24 hrs were also inoculated with parasites as described previously [(Niedelman et al. 2012)](https://paperpile.com/c/kMWCwc/loteh). The 24 well plates were then incubated undisturbed for 6 days at 37°C after which plaque areas were imaged and measured. For calculating the percentage of plaque loss, the following formula was used as described previously [(Niedelman et al. 2012, 2013)](https://paperpile.com/c/kMWCwc/loteh+jL9nZ) : [(Number of plaques in unstimulated condition - Number of plaques in stimulated condition)/Number of plaques in unstimulated condition] × 100. Plaque areas were captured and analyzed using a Nikon TE2000 inverted microscope equipped with Hamamatsu ORCA-ER digital camera, and NIS Elements Imaging Software, respectively. For all experiments, at least 20-25 plaques from technical duplicate wells were imaged.

**Measurement of NF-κB activation.** Activation of NF-κB was measured by measuring nuclear translocation of the NF-κB p65 subunit in HFFs using immunofluorescence or by using NF-κB reporter cells and measurement of luciferase activity as described previously [(Rosowski et al. 2011)](https://paperpile.com/c/kMWCwc/ujW9R). Briefly, HFFs were plated on coverslips in 24 well plates (1×10^5^ cells/well) and subsequently infected with Toxoplasma for 24 hrs. Following incubation, cells were fixed with 3% formaldehyde, permeabilized and blocked with a buffer containing 0.2% Triton X-100 along with 3% BSA and 5% goat serum. Cells were incubated with rabbit anti p65 (1:200 dilution, sc-109, Santacruz Biotechnology, CA, USA) overnight at 4 °C, after which each well was washed 3 times with 1x PBS, followed by incubation with goat anti rabbit Alexa fluor 594 (1:1000 dilution) and Hoechst 33258 (1:500 dilution) for 1 hr. Finally, coverslips were washed 5 times with 1× PBS and mounted with VECTASHIELD antifade mounting medium. For determination of nuclear translocation of NF-κB, nuclear intensity of at least 15 infected cells were measured for each experiment and coverslip.

**LDH release assay.** LDH release from culture supernatants of HFFs was measured as a marker of cell death according to the manufacturer’s protocol using mixtures of equal volume (100 µL) of culture supernatant and LDH reagent (Sigma Aldrich, St Louis, MO), incubation for 30 minutes and reading the absorbance at 490 nm using a plate reader. Confluent HFF cells in 96-well plates (2×104 cells/well) were stimulated with IFNγ (10 U/mL) for 24 hrs. Following that, cells were infected with Pru wild type and PruΔmag1 strains for another 24 hrs at 3 different MOIs (MOIs of 3, 5 and 7). To make comparisons between Pru strains, ‘real’ MOIs were matched by plaque assay [(Mukhopadhyay and Saeij 2020)](https://paperpile.com/c/kMWCwc/xW8lz).

***In vivo* infection for cytokine measurement**

Male and Female C57BL/6 mice that were 6–10 weeks old (The Jackson Laboratory) were used in the experiments. 10 mice (5 each of male and female) were kept per group. For i.p. infection, tachyzoites were grown *in vitro* in HFFs and extracted from host cells by passage through a 27- and 30-gauge needle, washed two times in PBS, and quantified with a hemocytometer. Parasites were diluted in PBS, and mice were inoculated i.p. with tachyzoites of each strain (5000 tachyzoites in 300 µl) using a 28-gauge needle. Animal experiments were performed in strict accordance with the recommendations in the Guide for the Care and Use of Laboratory Animals of the National Institutes of Health and the Animal Welfare Act, approved by the Institutional Animal Care and Use Committee at the University of California, Davis (UC Davis) (assurance number A-3433-01). To measure the *in vivo* cytokines, blood was collected from the tail vein of each mice at day 3 and serum samples were collected. IFNγ, IL-12p40, and TNFα levels were determined using commercially available matched pair ELISA kits (Invitrogen, Thermo Fisher Scientific) according to the manufacturer’s instructions.

**In vitro cytokine ELISA.** C57BL/6 BMDMs were seeded (10^5^ cells per well) in 96-well plates and left to adhere overnight at 37°C in 5% CO2. Cells were infected with freshly lysed tachyzoites of the different parasite strains at MOI = 3, 5 and 7, and supernatants (200 µl) were collected 24 hrs after infection. IL-12p40, IFNγ and TNFα levels were determined. To determine that cells were infected with equal numbers of viable parasites of the different strains, plaque assays were performed. Cytokine levels were measured using a commercially available matched pair ELISA kit (Invitrogen, Thermo Fisher Scientific) according to the manufacturer’s instructions.

**Determination of nitrite.** The Griess assay was used to determine Nitrite levels from culture supernatants of BMDMs stimulated with IFNγ (100 U/mL) and LPS (1 ng/mL) for 4 hrs and subsequently infected with indicated Toxoplasma strains for 20 hrs as described previously [(Jensen et al. 2011)](https://paperpile.com/c/kMWCwc/87n6i).

## Transmission electron microscopy

For *in vitro* bradyzoite differentiation, parasite strains were grown in HFF cells at pH 7 medium and 5% CO_2_ for 2 hours, then the medium were changed to differentiation medium and the parasite strains grew for 3 days at 0.5% CO_2_. After bradyzoite differentiation, cells were fixed for 1 hour with 2.5% glutaraldehyde, 2% paraformaldehyde in 0.1M sodium cacodylate buffer, post-fixed for 1 hour with 1% osmium tetroxide followed by 2% uranyl acetate, and then dehydrated through a graded series of ethanol using standard techniques. The samples were then embedded in LX112 resin (LADD Research Industries, Burlington VT). Ultrathin sections were cut on a Reichert Ultracut UCT, stained with uranyl acetate followed by lead citrate and viewed on a JOEL 1400EX transmission electron microscope at 120 kv.

[Iwano, Satoshi, Mayu Sugiyama, Hiroshi Hama, Akiya Watakabe, Naomi Hasegawa, Takahiro Kuchimaru, Kazumasa Z. Tanaka, et al. 2018. “Single-Cell Bioluminescence Imaging of Deep Tissue in Freely Moving Animals.” *Science* 359 (6378): 935–39.](http://paperpile.com/b/kMWCwc/IMM7u)

[Jensen, Kirk D. C., Yiding Wang, Elia D. Tait Wojno, Anjali J. Shastri, Kenneth Hu, Lara Cornel, Erwan Boedec, et al. 2011. “Toxoplasma Polymorphic Effectors Determine Macrophage Polarization and Intestinal Inflammation.” *Cell Host & Microbe* 9 (6): 472–83.](http://paperpile.com/b/kMWCwc/87n6i)

[Mukhopadhyay, Debanjan, and Jeroen P. J. Saeij. 2020. “Assays to Evaluate Toxoplasma-Macrophage Interactions.” *Methods in Molecular Biology*  2071: 347–70.](http://paperpile.com/b/kMWCwc/xW8lz)

[Niedelman, Wendy, Daniel A. Gold, Emily E. Rosowski, Joris K. Sprokholt, Daniel Lim, Ailan Farid Arenas, Mariane B. Melo, Eric Spooner, Michael B. Yaffe, and Jeroen P. J. Saeij. 2012. “The Rhoptry Proteins ROP18 and ROP5 Mediate Toxoplasma Gondii Evasion of the Murine, But Not the Human, Interferon-Gamma Response.” *PLoS Pathogens*. https://doi.org/](http://paperpile.com/b/kMWCwc/loteh)[10.1371/journal.ppat.1002784](http://dx.doi.org/10.1371/journal.ppat.1002784)[.](http://paperpile.com/b/kMWCwc/loteh)

[Niedelman, Wendy, Joris K. Sprokholt, Barbara Clough, Eva-Maria Frickel, and Jeroen P. J. Saeij. 2013. “Cell Death of Interferon-Gamma Stimulated Human Fibroblasts upon Toxoplasma Gondii Infection Induces Early Parasite Egress and Limits Parasite Replication.” *Infection and Immunity*, no. September (September). https://doi.org/](http://paperpile.com/b/kMWCwc/jL9nZ)[10.1128/IAI.00416-13](http://dx.doi.org/10.1128/IAI.00416-13)[.](http://paperpile.com/b/kMWCwc/jL9nZ)

[Rosowski, Emily E., Diana Lu, Lindsay Julien, Lauren Rodda, Rogier a. Gaiser, Kirk D. C. Jensen, and Jeroen P. J. Saeij. 2011. “Strain-Specific Activation of the NF-kappaB Pathway by GRA15, a Novel Toxoplasma Gondii Dense Granule Protein.” *The Journal of Experimental Medicine* 208 (1): 195–212.](http://paperpile.com/b/kMWCwc/ujW9R)
