# Supplementary material for: Toxoplasma gondii Matrix Antigen 1 Is a Secreted Immunomodulatory Effector
Source: mBio. 2021 May 18;12(3):e00603-21. doi: 10.1128/mBio.00603-21 (PMC8262993; doi:10.1128/mBio.00603-21)

Macro script used for segmentation

run("Duplicate...", " "); //need to preserve the original

run("Canny Edge Detector", "gaussian=3 low=2.5 high=7.5");

run("Create Selection"); //this is the area selection of the line

run("Enlarge...", "enlarge=4 pixel"); //enlarge it so that it doesn't leak for fill

setForegroundColor(255, 255, 255);

run("Fill", "slice"); //fill the inside of the line

run("Select None");

run("Fill Holes"); //binary -> fill the hole inside the white outline

run("Create Selection"); //this is PVM

run("ROI Manager...");

roiManager("Add"); //ROI=0, pvm

run("Enlarge...", "enlarge=-1"); //negative shrinks -1 um for cyst wall

roiManager("Add");//ROI=1, mtx

run("Enlarge...", "enlarge=6");// this gives 5 um host cell layer

roiManager("Add"); //ROI=2, hst

roiManager("Select", 0);

roiManager("Rename", "pvm");

roiManager("Select", 1);

roiManager("Rename", "mtx");

roiManager("Select", 2);

roiManager("Rename", "hst");

roiManager("Select", newArray(0,1));// pvm and mtx

roiManager("XOR"); //select between

roiManager("Add"); //this is cyst wall

roiManager("Select", 3);

roiManager("Rename", "csw");

roiManager("Select", newArray(0,2));//pvm and host

roiManager("XOR");//select between

roiManager("Add");//this is the host leak

roiManager("Select", 4);

roiManager("Rename", "hsl");

close();

roiManager("Select", newArray(0,1,2,3,4));

Example of segmentation


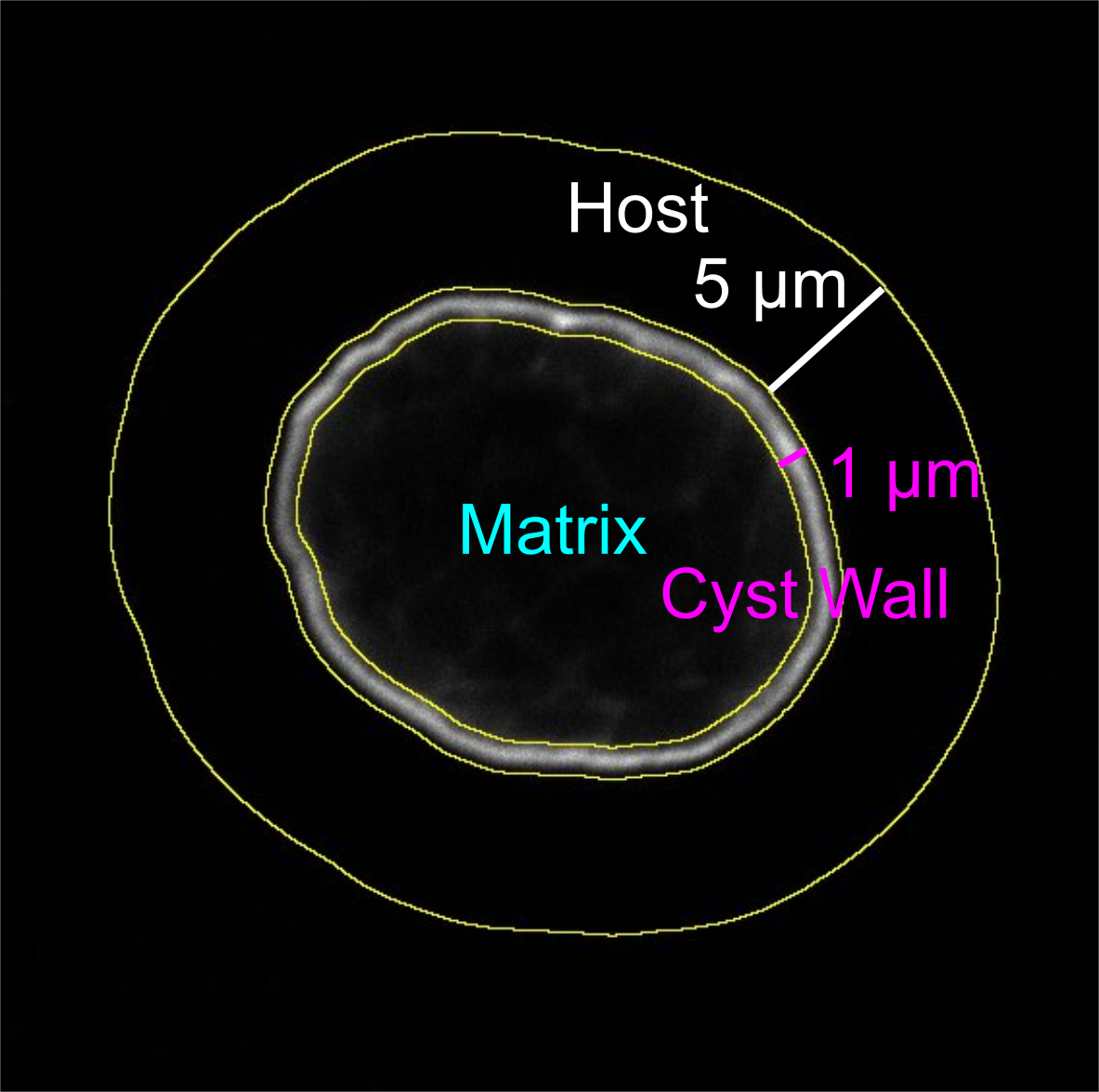

Supplement: DATA SET S2 [file mbio.00603-21-sd002.docx]
